# Progress Toward Poliovirus Containment Implementation — Worldwide, 2017–2018

**DOI:** 10.15585/mmwr.mm6735a5

**Published:** 2018-09-07

**Authors:** Jacqueline Fournier-Caruana, Nicoletta Previsani, Harpal Singh, Liliane Boualam, Joseph Swan, Anna Llewellyn, Roland W. Sutter, Michel Zaffran, MEng

**Affiliations:** ^1^Polio Eradication, World Health Organization, Geneva, Switzerland; ^2^Center for Global Health, CDC.

Substantial progress has been made since the World Health Assembly (WHA) resolved to eradicate poliomyelitis in 1988 (*1*). Among the three wild poliovirus (WPV) types, type 2 (WPV2) was declared eradicated in 2015, and type 3 (WPV3) has not been reported since 2012 (*1*). In 2017 and 2018, only Afghanistan and Pakistan have reported WPV type 1 (WPV1) transmission (*1*). When global eradication of poliomyelitis is achieved, facilities retaining poliovirus materials need to minimize the risk for reintroduction of poliovirus into communities and reestablishment of transmission. Poliovirus containment includes biorisk management requirements for laboratories, vaccine production sites, and other facilities that retain polioviruses after eradication; the initial milestones are for containment of type 2 polioviruses (PV2s). At the 71st WHA in 2018, World Health Organization (WHO) Member States adopted a resolution urging acceleration of poliovirus containment activities globally, including establishment by the end of 2018 of national authorities for containment (NACs) to oversee poliovirus containment (*2*). This report summarizes containment progress since the previous report (*3*) and outlines remaining challenges. As of August 2018, 29 countries had designated 81 facilities to retain PV2 materials; 22 of these countries had established NACs. Although there has been substantial progress, intensification of containment measures is needed.

## Background

The Global Polio Eradication Initiative continues to make progress toward polio eradication. Only 22 cases from a single serotype (WPV1) were reported in 2017 from Afghanistan and Pakistan, two of the three countries with endemic poliovirus transmission (*1*). Nigeria did not detect WPV cases in 2017 (*1*).

The last reported indigenous WPV2 case was detected in 1999 (*1*). After the global certification of WPV2 eradication in 2015, the type 2 vaccine component was synchronously withdrawn from use worldwide in May 2016 by switching from trivalent oral poliovirus vaccine (tOPV, containing vaccine virus types 1, 2, and 3) to bivalent OPV (bOPV, containing types 1 and 3). Although the global switch was implemented without major issues in most countries, the detection of vaccine-derived poliovirus type 2 (VDPV2) (poliovirus strains that have mutated from the vaccine virus and reverted to neurovirulence because of unusually prolonged circulation in populations with low immunity levels) has necessitated the distribution of 126 million doses of monovalent type 2 OPV (mOPV2) for outbreak control in 11 countries (Cameroon, Chad, Democratic Republic of the Congo, Ethiopia, Kenya, Mozambique, Niger, Nigeria, Pakistan, Somalia, and Syria) (*1*,*4*,*5*) (Figure). In 2018, outbreaks in the Democratic Republic of the Congo, the Horn of Africa (Ethiopia, Kenya, and Somalia), and northern Nigeria have required further use of the mOPV2 global stockpile.*

**FIGURE:**
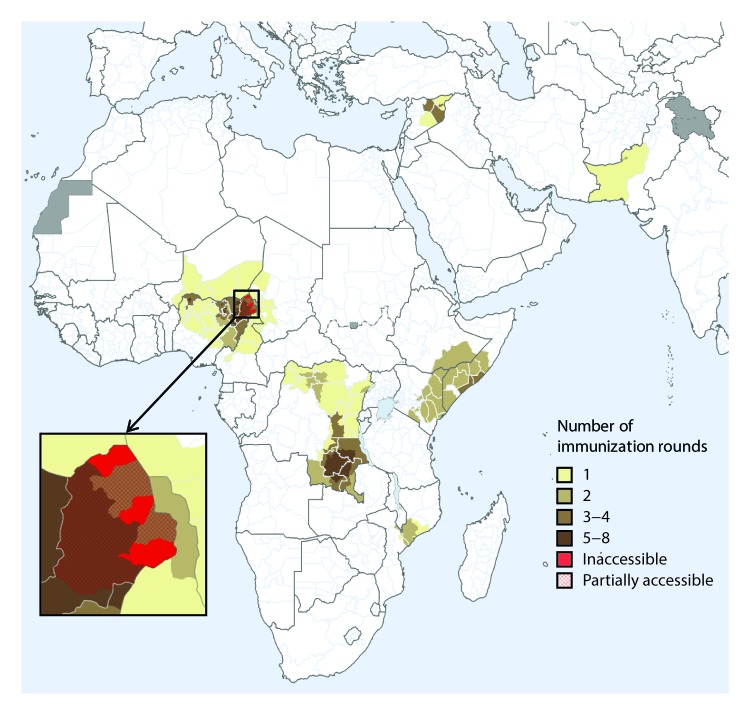
Areas where monovalent oral poliovirus vaccine type 2 (mOPV2) has been used for prevention and control of circulating vaccine-derived poliovirus type 2 transmission,* by number of immunization rounds — Worldwide, 2016–2018^†^ * In Mozambique, mOPV2 was used in response to a type 2 ambiguous vaccine-derived poliovirus (a vaccine-derived poliovirus isolate from a person with or without acute flaccid paralysis and with no known immunodeficiency, or from environmental samples, without evidence for circulation). ^†^ Data as of August 8, 2018, and subject to change.

Since the global withdrawal of type 2-containing OPVs in 2016, PV2 (WPV2, VDPV2, and OPV2) must be retained under stringent containment conditions (*6*). Containment is intended to minimize the risk for release of polioviruses from facilities, which would permit occurrence of paralytic disease and reestablishment of transmission.

## Guidance and Oversight

The WHO Global Action Plan to minimize poliovirus facility–associated risk after type-specific eradication of wild polioviruses and sequential cessation of oral poliovirus vaccine use (GAPIII) (*7*) defines the biorisk management standards to be followed by facilities retaining poliovirus materials. Implementation of these standards begins with the establishment of national inventories for facilities retaining PV2 materials. The Containment Certification Scheme to support the WHO Global Action Plan for Poliovirus Containment (GAPIII-CCS) (*8*) defines the recommended mechanisms for verifying compliance with global poliovirus containment requirements within poliovirus essential facilities (PEFs). The implementation of poliovirus containment is complicated by the potential for facilities to retain materials that might incidentally contain polioviruses (e.g., biological specimens such as fecal, respiratory, or sewage samples collected at a time and place where WPVs were circulating or where OPV2 was in use). To help facilities identify, eliminate, and minimize risks for handling and storing such samples, WHO in 2018 issued guidance to minimize risks for facilities that collect, handle, or store materials potentially containing infectious polioviruses (*9*). Facilities with a high probability of handling or storing such samples include those working with enteric disease agents (e.g., rotavirus, *Salmonella*, or hepatitis viruses), respiratory disease agents (e.g., influenza virus, *Mycobacterium tuberculosis*, or measles virus), or that are involved with nutrition research or environmental studies. All 194 WHO Member States are requested to implement the guidance and complete reports on PV2 material by April 2019.

Infrastructure and mechanisms for poliovirus containment governance were established to support national and global containment implementation and certification processes.^†^ At the 71st WHA in May 2018, WHO Member States unanimously adopted Resolution WHA71.16 (*2*), which urged international commitment to expedite full implementation of GAPIII requirements worldwide. After adoption of the resolution, countries are expected to complete PV2 inventories, destroy unneeded PV2 materials, and begin inventories for WPV1 and WPV3 materials in accordance with WHO guidance. In addition, countries must reduce to a minimum the number of facilities designated to retain polioviruses, appoint a NAC by the end of 2018, and formally engage these designated PEFs in the containment certification process no later than the end of 2019.

The Global Commission for the Certification of Eradication of Poliomyelitis (GCC) is the oversight body for containment until the certification of global eradication of poliomyelitis. The GCC Containment Working Group reviews PEF containment certification applications submitted by NACs to ensure that GAPIII requirements are met, according to the GAPIII-CCS process. The Containment Advisory Group serves as the advisory body to the Director-General of WHO and issues regular reports on technical issues related to the implementation of GAPIII.^§^ The Strategic Advisory Group of Experts on immunization provides recommendations on polio immunization policies and coverage targets in accordance with the population immunity requirements (secondary safeguards) of GAPIII (*10*). The Containment Management Group manages and coordinates GPEI partner support of global containment activities, including the recent development of a framework for containment risk assessment and risk ranking for PEFs. In October 2018, the Expert Committee on Biological Standardization is expected to endorse the revised WHO Technical Report Series 926, which provides guidelines for the safe production and quality control of poliomyelitis vaccines in the containment era.^¶^ The revised Technical Report Series 926 and GAPIII will be closely aligned.

## Progress

To prevent reintroduction of poliovirus and reestablishment of transmission, the number of facilities designated to retain PV2 materials will need to be reduced to the minimum necessary to perform critical national and international functions (e.g., vaccine production, diagnosis, and research). However, early counts of designated PEFs included well over 100 facilities worldwide. To address this issue, country visits have been made, and governments will continue to be urged to carefully consider the implications of designating facilities to retain poliovirus materials and, where needed, to encourage the establishment of NACs. As of August 2018, a total of 81 PEFs had been designated by governments in 29 countries to retain PV2 materials, including 22 that had reported the establishment of their NACs (Table), compared with reports from 2017, when 86 facilities within 30 countries were planning to move forward with PEF designation and only 18 NACs had been established (*3*).

**TABLE:** Number of designated poliovirus-essential facilities (PEFs) retaining poliovirus type 2 (PV2)* and National Authorities for Containment (NACs), by World Health Organization (WHO) regions — Worldwide, 2018^†^

WHO region	No. of countries	No. of NACs	No. of PEFs	Type of PV2 materials retained			Type of facility		
				WPV2	Both WPV2/VDPV2 and OPV2/Sabin2	Only OPV2/Sabin2	Salk-IPV^§^ production sites	Sabin-IPV^§¶^ production sites	Diagnostic or research laboratories
AFR	1	1	1	0	1	0	0	0	1
AMR	6	5	19	7	4	8	1	0	18
EMR	2	2	2	0	0	2	0	1	1
EUR	13	8	40	7	23	10	5	2	33
SEAR	2	2	3	1	0	2	0	1	2
WPR	5	4	16	0	4	12	0	11	5
**Total**	**29**	**22**	**81**	**15**	**32**	**34**	**6**	**15**	**60**

**Abbreviations**: AFR = African Region; AMR = Region of the Americas; EMR = Eastern Mediterranean Region; EUR = European Region; IPV = inactivated polio vaccine; OPV2 = type 2 oral poliovirus vaccine; SEAR = South-East Asia Region; VDPV2 = type 2 vaccine–derived poliovirus; WPR = Western Pacific Region; WPV2 = type 2 wild poliovirus.

* Includes WPV2/circulating VDPV2 and OPV2/Sabin2 (attenuated live type 2 poliovirus strains used in oral vaccines, including vaccine seed stocks and infectious vaccine production materials as well as viruses closely related to attenuated vaccine viruses recovered from fecal and respiratory specimens or sewage samples).

^†^ Data as of August 28, 2018, and subject to change.

^§^ Salk-IPV is a vaccine made from wild poliovirus strains that are inactivated; Sabin-IPV is an inactivated vaccine made from attenuated live poliovirus strains.

^¶^ Includes potential future producers in different clinical and preclinical phases of Sabin-IPV development.

NACs are the authorities for auditing facilities and issuing containment certificates. This three-certificate process is overseen by the GCC through the Containment Working Group and includes a certificate of participation (i.e., official recognition as a PEF), interim certificate of containment (i.e., indication of not achieving all GAPIII requirements during the PV2 period while addressing the need for full compliance with GAPIII), and certificate of containment (i.e., full compliance with GAPIII requirements). To date, the GCC has endorsed one certificate of participation submitted according to the GAPIII-CCS process by the NAC of Sweden.** All remaining applications for certificate of participation must be submitted for the approval by the GCC Containment Working Group by the end of 2019.

To strengthen the auditing capacity of NACs and to create a pool of international GAPIII auditors, WHO continues to provide biorisk management and GAPIII auditor trainings throughout the six WHO regions. In addition, WHO and global partners are providing regional and national level training in the implementation of the guidance for retaining potentially infectious poliovirus materials.

## Discussion

Substantial progress toward poliovirus containment has been made during 2017–2018, including reduction in the number of designated PEFs, establishment of the majority of NACs, and the initiation of the containment certification process. In addition, WHO and global partners have implemented containment trainings in all six WHO regions, global polio advisory groups have made recommendations to facilitate GAPIII implementation, and the 2018 WHA resolution has prioritized global poliovirus containment.

Poliovirus containment is a national responsibility, and it is expected that the number of designated PEFs will be further reduced as countries carefully determine whether the programs, funding, and other resources needed to achieve and maintain full compliance with GAPIII requirements are a national or international priority. The implementation of containment activities, such as completion of national PV2 inventories, establishment of NACs, and certification of PEFs, has required more time than had been anticipated. This extended timeline resulted from many factors, including delayed submission of PV2 inventories, training and retention of qualified GAPIII-CCS auditors, and extensive PEF preparations required to meet GAPIII standards. In addition, many countries do not have established legislation to provide NACs with legal authority to enforce GAPIII, or existing national biosafety regulations are not consistent with GAPIII requirements. The identification of potentially infectious poliovirus materials in nonpolio facilities is an enormous global undertaking that places a burden on facilities that were never intended to handle polioviruses. Continued global engagement, education, and technical body oversight; a robust communication strategy; and enhanced political will are required to resolve these issues.

Poliovirus containment is a global effort that is integral to polio eradication. In the coming year, all countries are expected to complete their inventories and destroy or transfer unneeded PV2 materials and initiate the inventory and destruction or transfer of unneeded WPV1 and WPV3 materials. Countries also need to carefully weigh the risks and benefits of designating facilities for the retention of poliovirus materials and the need to comply with the primary (facility), secondary (population immunity), and tertiary (sanitation and hygiene) safeguards. Importantly, countries hosting PEFs must establish NACs and begin the certification process following the GAPIII-CCS process. Further intensification of measures will be needed to ensure that effective containment will be in place by the time the world is certified polio-free.

**Summary**

**What is already known about this topic?**

Wild poliovirus type 2 was certified eradicated in 2015. All type 2 polioviruses must now be destroyed or safely and securely contained to minimize the risk for reintroduction into communities and reestablishment of transmission.

**What is added by this report?**

Twenty-nine countries have designated 81 facilities for the retention of needed poliovirus type 2 materials to perform critical national or international functions under certified conditions, including vaccine production, diagnosis, and research.

**What are the implications for public health practice?**

If not securely contained, release of the virus could result in reestablishment of endemic or epidemic poliovirus transmission. Further measures will be needed to ensure effective containment by the time the world is certified polio-free.

## Footnotes

* http://polioeradication.org/polio-today/polio-now/this-week.

† http://polioeradication.org/who-we-are/governance-and-structure.

§ http://polioeradication.org/tools-and-library/policy-reports/advisory-reports/containment-advisory-group.

¶ http://www.who.int/biologicals/BS.2018.2350_Poliomyelitis_vaccine_Guidelines.pdf.

** http://polioeradication.org/news-post/sweden-takes-important-first-step-to-demonstrate-containment-of-type-2-poliovirus.
